# *BARE* Retrotransposons Are Translated and Replicated via Distinct RNA Pools

**DOI:** 10.1371/journal.pone.0072270

**Published:** 2013-08-06

**Authors:** Wei Chang, Marko Jääskeläinen, Song-ping Li, Alan H. Schulman

**Affiliations:** 1 Institute of Biotechnology, Viikki Biocenter, University of Helsinki, Helsinki, Finland; 2 Genome-Scale Biology Program, University of Helsinki, Biomedicum, Helsinki, Finland; 3 Biotechnology and Food Research, MTT Agrifood Research Finland, Jokioinen, Finland; Lady Davis Institute for Medical Research, Canada

## Abstract

The replication of Long Terminal Repeat (LTR) retrotransposons, which can constitute over 80% of higher plant genomes, resembles that of retroviruses. A major question for retrotransposons and retroviruses is how the two conflicting roles of their transcripts, in translation and reverse transcription, are balanced. Here, we show that the *BARE* retrotransposon, despite its organization into just one open reading frame, produces three distinct classes of transcripts. One is capped, polyadenylated, and translated, but cannot be copied into cDNA. The second is not capped or polyadenylated, but is destined for packaging and ultimate reverse transcription. The third class is capped, polyadenylated, and spliced to favor production of a subgenomic RNA encoding only Gag, the protein forming virus-like particles. Moreover, the *BARE2* subfamily, which cannot synthesize Gag and is parasitic on *BARE1*, does not produce the spliced sub-genomic RNA for translation but does make the replication competent transcripts, which are packaged into *BARE1* particles. To our knowledge, this is first demonstration of distinct RNA pools for translation and transcription for any retrotransposon.

## Introduction

Long terminal repeat (LTR) retrotransposons or Class I transposable elements are ubiquitous in the eukaryotes and can comprise over 80% of the large genomes of plants [1,2]. They propagate similarly to the intracellular phase of retroviruses: by a “copy and paste” cycle of transcription of genomic copies, translation, packaging of transcripts into virus-like particles (VLPs) composed of Gag, reverse transcription, and targeting of the cDNA copy to the nucleus for integration into the genome [3,4]. The lifecycle depends upon proteins encoded by the retrotransposon itself.

The structural Gag is often in a separate open reading frame (ORF) from *Pol*, which encodes the enzymes reverse transcriptase (RT), RNAse H (RH), aspartic proteinase (PR), and integrase (IN). The stoichiometry between *gag* and *pol* products is critical for replication because the assembly of the VLP requires excess Gag relative to the enzymes [5,6]. A common strategy among retroviruses and retrotransposons to produce more Gag is -1 or +1 translational frameshifting between *gag* and *pol* [6–8]. However, the *Copia* superfamily of retrotransposons [3] and also most plant retrotransposons have only a single ORF [9]. While alternative splicing in *copia* of *Drosophila* deletes virtually all of *pol*, 2950 nt, generating an RNA dedicated to Gag translation [10], this has not been seen for other members of the superfamily. In some cases post-translational protein degradation serves to achieve a molar excess of Gag [11].

Another major conundrum is that reverse transcription of retrotransposon and retrovirus RNA, which destroys the RNA template, conflicts with its further translation [12]. Alternatively, instead of a single pool of RNA, separate populations may serve each function with not all RNA being sequestered into capsids for reverse transcription. Among the retroviruses, Murine Lukemia Virus (MLV) may use distinct RNA pools [13], whereas HIV-1 and -2 do not [14]. The question has not been investigated for retrotransposons.

Although retrotransposons comprise much of most plant genomes, the details of their lifecycle have been investigated for only a few. The *BARE* retrotransposon of superfamily *Copia* accounts for over 10% of the barley genome [2,15]. *BARE1* has no frameshift between *gag* and *pol* [16,17]. A variant called *BARE2* cannot express Gag [16,18]. *BARE1* and *BARE2* produce multiple classes of RNA transcripts from two TATA boxes, of which only 15 to 25% are polyadenylated [19]. Moreover, those which are polyadenylated lack the R domain needed for reverse transcription. These observations raise the questions addressed here: which *BARE* RNAs serve for translation, which ones are packaged, and does *BARE* use an alternative to frameshifting for Gag production. We were able to demonstrate not only different RNA pools for translation and reverse transcription but also a novel splicing pattern for Gag synthesis.

## Materials and Methods

### Plant Materials and RNA Isolation

Barley (*Hordeum vulgare* L.) plant materials and callus cultures used for RNA isolation, as well as the methods used to isolate the RNA, are described in the Materials S1 *.*

### RNA End Structure

The presence of a 5’ 7-methylguanosine cap on *BARE* transcripts was assayed by the procedure called RLM-mediated rapid amplification of 5’ cDNA ends (5’ RLM-RACE) using a kit (FirstChoice®RLM-RACE, Ambion AM1700) with small changes and a custom adapter. To examine 3’ polyadenylation of polyribosome-associated RNA, 3’ RLM-RACE was carried out. Details of both methods are presented in the Materials S1.

### Analysis of *BARE1* and *BARE2* Expression Levels

To evaluate the relative expression levels of *BARE1* and *BARE2*, RT-PCR was carried out using primer AP4 (Table S1) to prime cDNA synthesis and then primers LS1 (Table S1) and AP4 for the amplification reaction. The primer pair binds both to *BARE1* and *BARE2*, and amplifies them equally well [16]. The two retrotransposon families were amplified from genomic DNA using the same primer pair.

### Polyribosome Isolation and RT-PCR

Barley callus cultures cells were collected, frozen under liquid N_2_, and then pulverized with mortar and pestle under liquid N_2_. Polyribosomes were isolated largely as previously described [20]. The procedure is described in detail in the Materials S1.

### Splicing assays

Splicing analysis was made with 1µg of RNA treated twice with DNase, reverse transcribed into cDNA as using the *BARE*-specific primer 81567. The *BARE* transcripts were amplified following cDNA production using two primers close to the spliced region, F1593 and F1594 (Table S1), and a PCR program consisting of 94^o^C for 5 min, 40 cycles of 94^o^C for 30 sec, 56^o^C for 1 min, and 72^o^C for 1 min, with a final extension at 72^o^C for 5 min. To investigate splicing in polyadenylated RNA, primer 81567 was used to initiate cDNA synthesis, followed by F1594 and AP4 for PCR amplification. For detection of capped RNA splicing, an RNA linker was first ligated to dephosphorylated and decapped RNA and then cDNA synthesized as above. Two PCR reactions were prepared by 5’ RACE using the linker and 81567 as the primer pair. One reaction was amplified, the other served as a control. The second RACE reaction used 1µl of either the first PCR product or the control as the template and primers F1594 and 81567. Controls produced no signal from the second amplification. The decapped RNA gave the same size product as did total RNA RT-PCR using same primer pair.

## Results

### *BARE1* but not *BARE2* RNA Is Spliced

**Figure 1:**
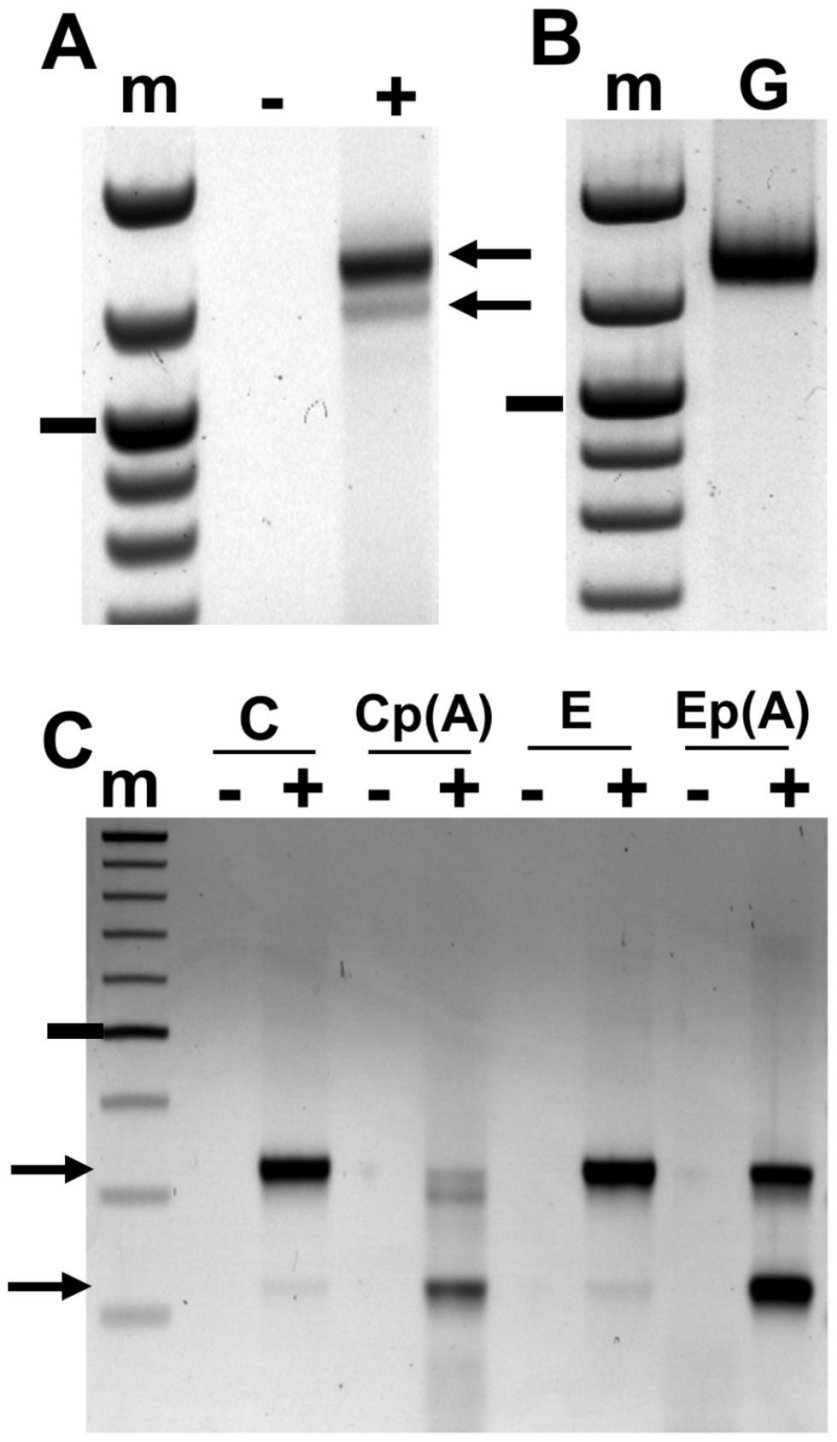
Splicing of *BARE* transcripts. **A**. Agarose gel electrophoresis of the RT-PCR amplification product from callus RNA using primers LS1 and AP4. The upper band (arrow, 1.3 kb) contains products from both *BARE1* and *BARE2* and is the same size as the amplification product from genomic DNA using the same primer pair (B); the lower, faint band (A, arrow, 1.2 kb) is the spliced *BARE1* form and it is not seen in the genomic DNA amplification. **B**. RT-PCR (+) from total RNA (primers LS1 and AP4); the lanes display reactions containing reverse transcriptase (+), negative control lacking reverse transcriptase (-), or genomic DNA instead of RNA (G) as the positive control. **C**. Detection of splicing in embryo, E, and callus, C, total and poly(A) RNA (labelled p (A)) using a *BARE1*-specific primer pair (81567, F1594). Arrows indicate the unspliced and spliced forms. Size markers (m), 100 bp ladder, marked band is 1 kb (A, B) or 0.5 kb (C).

**Figure 2:**
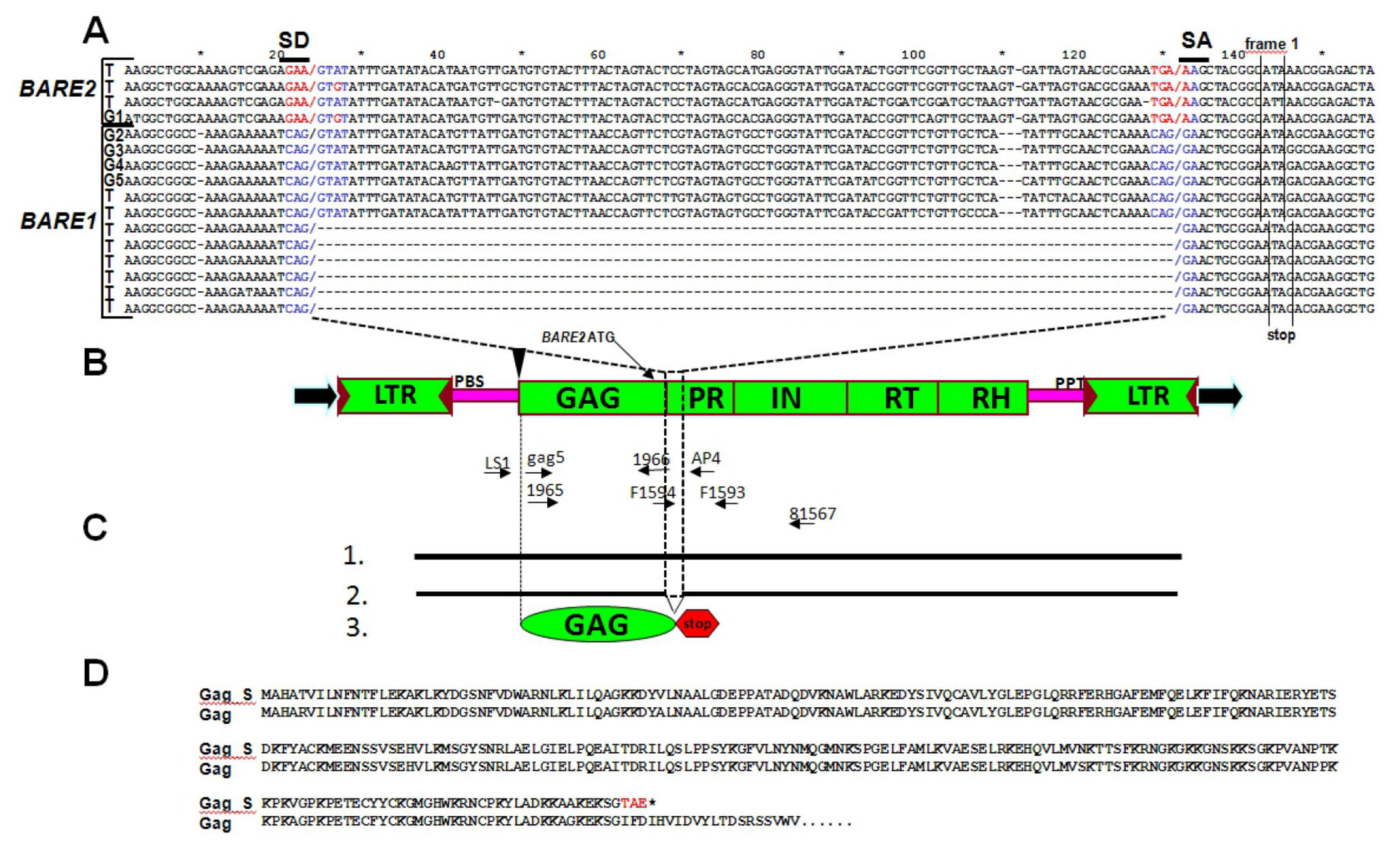
Splicing of *BARE1*. **A**. Alignment of a set of *BARE* genomic DNA sequences and cDNA clones showing the forms with the consensus splice junctions (SD and SA) within the *gag* domain. Nucleotides shaded blue match the consensus slice junctions, those in red do not. Genomic DNA sequences are labelled as “G”, cDNA as “T”. Genomic sequences in the alignment are: G1, AJ279072; G2, Z17327; G3, AY66155; G4, AY485643; G5, BQ900685. **B**. Schematic diagram of *BARE* retrotransposons showing the LTRs, encoded proteins of the open reading frame, and the cDNA priming sites (PBS, PPT), together with the position of the diagnostic primers as arrows below. The inverted triangle indicates the 8 bp deletion of the start codon in *BARE2* that eliminates synthesis of Gag; the following ATG for the *Pol* domain is indicated. **C**. Diagrams of the unspliced (1) and spliced (2) forms of the *BARE1* transcripts as well as the translated product of the spliced form (3). **D**. Conceptual translation of the *BARE1* ORF (Accession Z17327) covering the Gag and part of the PR region for the unspliced (Gag) and spliced (Gag_S) transcript forms. Amino acids altered by the splice-induced frameshift are shown in red, the stop codon as *.

Amplification of *BARE* from total RNA produced two products, one consistent with the size of genomic *BARE* copies and the other somewhat smaller (Figure 1). Amplifications from genomic DNA (Figure 1B) produced no smaller product. A smaller product was amplified from the RNAs of all tissues tested, which were callus and embryo (Figure 1C) as well as leaf and root (data summarized in Table 1). A total of 60 clones were sequenced from the more abundant, longer product; *BARE1* and *BARE2* were equally present among the sequences. The two larger products seen in callus RNA (Figure 1C) differ only by amplification from a secondary PCR priming site. All the sequences from the short product, however, were from *BARE1* and contained a deletion at the beginning of pr domain, comprising a segment of 104 nt flanked by GT and AG respectively at the left and right borders (Figure 2A).

**Table 1:** Summary of *BARE* RNA species detected.

**TE**	**Structure**				**Occurrence**		
	**Sp**	**Cap**	**pA**	**R**	**C**	**L**	**E**
*BARE1*	+	+	+	-	+	+	+
*BARE1*	-	+	+	-	+	+	+
*BARE1*	-	-	-	+	+	+	+
*BARE2*	-	-	-	-	+	+	+
*BARE2*	-	+	+	-	+	+	+

Abbreviations: TE, retrotransposon family; Sp, transcript spliced; Cap, cap(Gppp) present; pA, polyadenylation; R, R-domain present; C, callus; L, leaf; E, embryo Each table row corresponds to one RNA type having the features marked as present (+) or absent (-)

The presence of the short form of *BARE1* in the RNA but not the genome suggested that it is a spliced transcript. The *BARE1* genomic sequences contain conserved CAG/GTAT and CAG/GA motifs respectively matching the 5’ and 3’ junctions CAG/---/GA that flank the segment missing in the minor cDNA sequence (Figure 2A). These are a very good match to the consensus sequence AG/GT for the donor site in a genome-wide survey of the model species *Brachypodium* *distachyon* [21] and for consensus donor and acceptor splice sites, respectively C(A) AG/GTA and CAG/G, in *Arabidopsis* and rice [22,23]. The *BARE1* junctions are also well identified by the Netgene2 splice site predictor (http://www.cbs.dtu.dk/services/NetGene2/) within the *Arabidopsis* genome and by SplicePredictor (http://deepc2.psi.iastate.edu/cgi-bin/sp.cgi) against maize and human genomes, supporting the interpretation that the shorter *BARE* transcript is a splicing product.

Notably, the predicted splicing signals are not found in the *BARE2* genomic or RNA sequences (Figure 2A). The areas immediately 5’ to the donor site and 3’ to the acceptor site of *BARE1* are also divergent in *BARE2*, although the sequence of the facultative intron is quite similar in both. While the short form comprised a minor fraction of total RNA comprising both *BARE1* and *BARE2*, about 12.5% using the primers (Figure 1A) that amplified both, it represented fully half of the *BARE1*-specific product amplified from polyadenylated RNA (Figure 1C). The predicted and sequenced splice junction is 2 nt beyond the end of the *gag* coding domain [16,17], thereby creating a stop codon three amino acids beyond the end of Gag (Figure 2B,C,D) followed by many more within *pol*. Consequently, the spliced RNA can express only Gag (Figure 2D). The predicted molecular weight of the Gag from the spliced RNA is 32.1 kDa, the same as predicted from the electrophoretic mobility of Gag from VLPs [18].

Introns in plants are generally 15% more U-rich than the flanking exons, while exons are 15% more GC-rich than the corresponding intron [24–26]. In *BARE1*, the 52 nt flanking the slicing signals are 13.5% U vs. 37.5% U for the 104 nt intron, making the intron 24% and 2.8-fold more U-rich than the exon segments. In *BARE2*, which does not splice, the intron region is only 16.7% and 1.9-fold more U-rich than the surrounding region. Moreover, these flanking exon regions in *BARE1* are GC-rich, being 53.8% GC, 16.3% more GC than the intron, whereas the corresponding *BARE2* segment is 47.2% GC, only a 7.6% difference with the surrounding regions. Both these measures and the splice site comparison show that the *BARE1* intron conforms to expectations for plant intron functionality and suggest that there has been selective pressure on *BARE1* for splicing compared with *BARE2.*

### Transcripts from BARE TATA1 are uncapped, but those from TATA2 are capped

**Figure 3:**
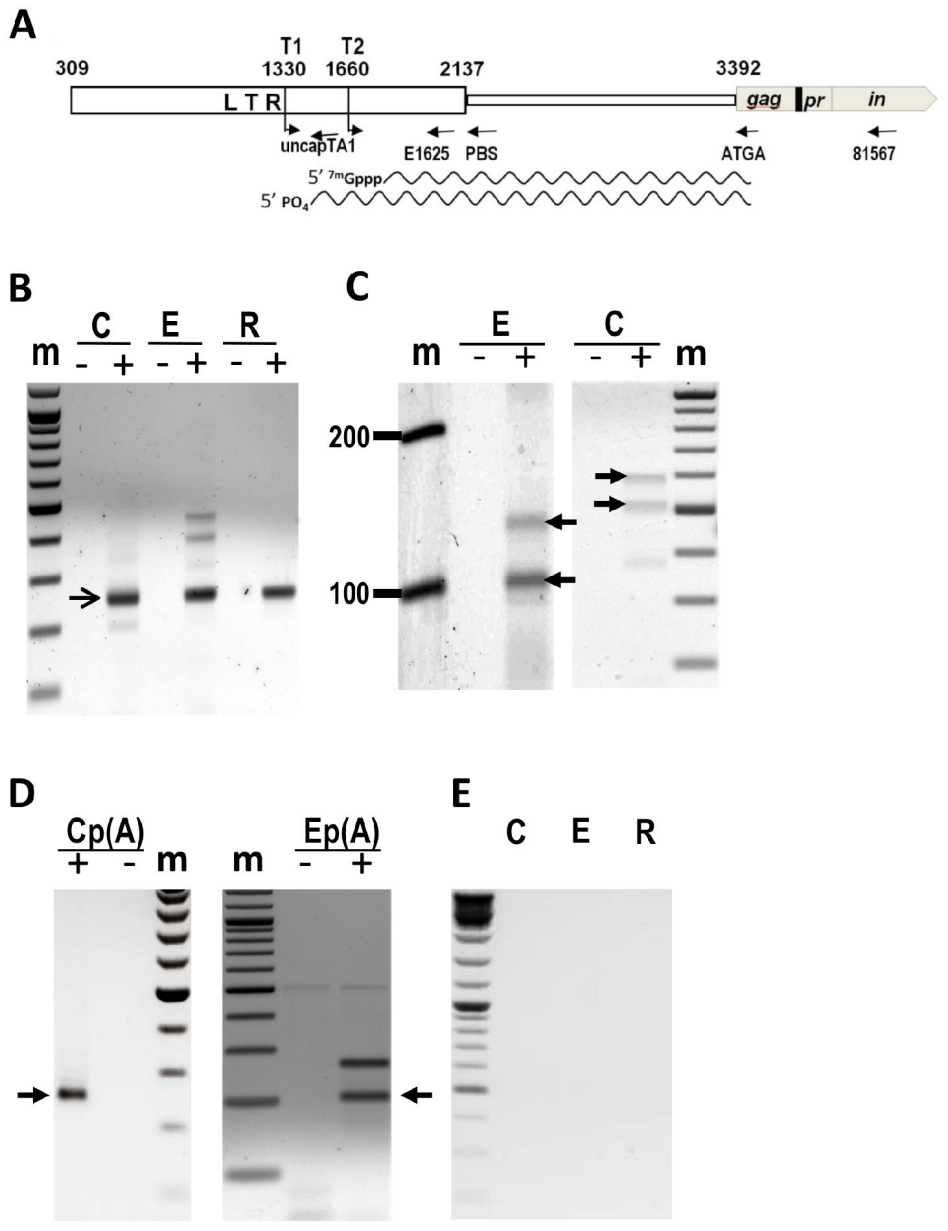
Capping of *BARE* transcripts. **A**. Schematic representation of the *BARE* LTR and part of the ORF. The black box between *gag* and pr represents the specific deletion in *BARE2* (deletion not to scale). The 5’ LTR is shown as a thick box, the region between the LTR and the start codon of *gag* as a thin box. The position of TATA1 (T1) and TATA2 (T2) are marked with bent arrows, their position and those of the beginning and end of the LTR and the beginning of *gag* numbered according to acc. Z17327. Primers used for PCR and making cDNA are indicated by arrows below. Wavy lines indicate the capped TATA2 and uncapped TATA1 transcripts identified by RLM-RACE PCR. **B**. RLM-RACE PCR analysis of the transcription start sites of *BARE* from total RNA in different tissues (C, callus; E, embryo; R, root) following phosphatase and pyrophosphatase treatment to select for capped transcripts. (+) and (-) indicate the presence or absence (control) of reverse transcriptase in the assay. The product size (arrow, 244 bp) corresponds to amplification from the 5’ adapter primer and E1625. **C**. Detection of uncapped *BARE* transcripts in embryos and callus total RNA by RLM-RACE PCR without phosphatase and pyrophosphatase treatment. The larger band (arrow) corresponds to *BARE1*, the smaller (arrow) to *BARE2*. **D**. Detection of capped *BARE* poly(a) RNA (arrow) in callus (Cp(A)) and embryo (Ep(A)) by RLM-RACE PCR; RNA treated as in (B). The upper band in embryo is due to a secondary priming site; the amplification generates a product of the same size for *BARE1* and *BARE2*. 100 bp ladders (m) are shown. **E**. Control reactions treated with phosphatase but not subsequent pyrophosphatase.

Because the two RNA splice variants of *BARE1* possess different translation capacities, we investigated the transcripts for features associated with translation. The RNA destined for translation in eukaryotic cells commonly receives a 7-methylguanosine cap as part of the maturation process [27], although many plant viruses as well as HIV exploit cap-independent translation instead [28,29]. We earlier showed [19] that *BARE* produces ten classes of transcripts from two TATA boxes (Figure 3A), five each from TATA1 and TATA2. In order to investigate which might be translated, we first looked at those which have 5’ caps.

Capped and uncapped RNAs were distinguished by enzymatic treatment before RNA-ligase-mediated (RLM) PCR, respectively by pyrophosphatase decapping and phosphatase 5’ dephosphorylation. Pyrophosphatase-mediated cap removal generates a 5’ phosphate in its place, which will allow ligation of an RNA adapter and PCR. Uncapped 5’ ends can be ligated directly without pyrophosphatase treatment.

**Figure 4:**
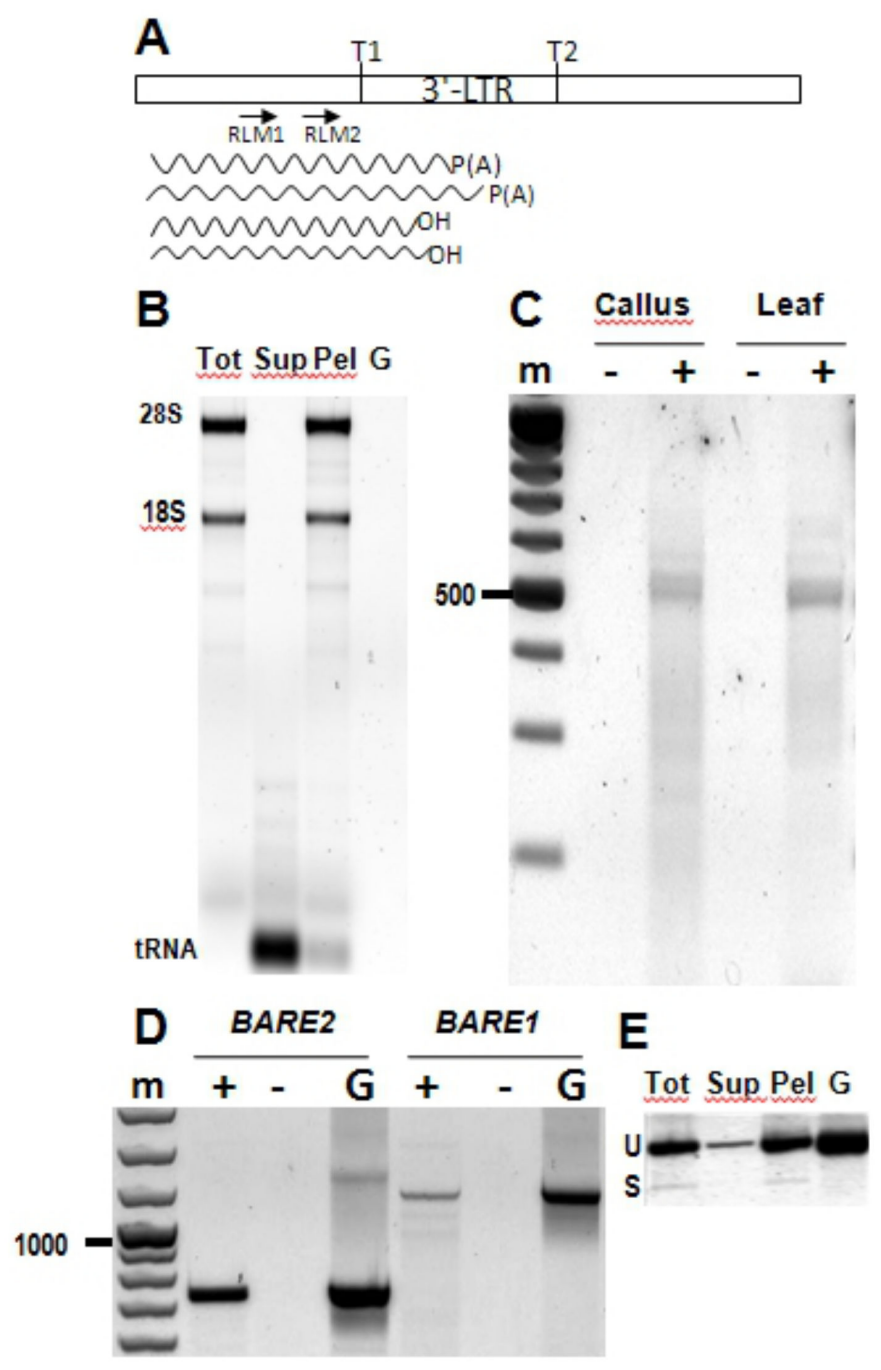
Spliced *BARE* RNA is associated with polyribosomes. **A**. Diagram of the 3’ LTR, indicating for reference the position of the two TATA boxes TATA1 (T1) and TATA2 (T2); only those in the 5’ LTR serve to transcribe the retrotransposon. The positions of forward primers RLM1 and RLM2 for 3’ RLM-RACE are shown. The wavy lines show, respectively, the approximate termination positions of the polyribosome-associated poly(A) RNA. **B**. Supernatant (Sup) and polyribosome pellet (Pel) fractions from total callus RNA (Tot) ultracentrifuged on a 10-45% sucrose gradient. rRNA bands are labelled. **C**. Electrophoresis of RLM-RACE reactions from polyribosome-associated callus and leaf RNA amplifying both *BARE1* and *BARE2*. A 100 bp size ladder (m) is shown, 500 bp marked. **D**. Amplification of *BARE2* and *BARE1* from polyribosome-associated callus RNA (primers 1965 and 1966 for *BARE2*, primers Gag5 and AP4 for *BARE1*, Figure 2). Negative controls (-) for the presence of genomic DNA contamination lack reverse transcriptase; positive controls, G, contain genomic DNA. Bars point to 1000 bp and 500 bp size markers, m. **E**. RT-PCR assay (primers F1594 and F1593, Figure 2, which are not specific to *BARE1*) from the fractions in (B). For size comparison, PCR from genomic DNA with the same primers is shown on the right. Unspliced and spliced transcripts are indicated as U and S, respectively. The RT-minus controls gave no amplification.

The experiments were carried out with primers positioned (Figure 3A) so that RNA products of both TATA1 and TATA2 could be detected, but only those from intact *BARE* elements containing internal domains and not from read-through transcripts of solo LTRs. Capped RNAs transcribed from *BARE* were found in all tissues examined (Figures 2, 4B). Control reactions lacking pyrophosphatase gave no PCR product (Figure 3E). Sequenced PCR products showed that capped transcripts derive only from TATA2; the longer products from the embryo (Figure 3B) were sequenced and are non-specific. The capped transcripts start at nt 1686-1689 (Z17327), corresponding with the published RACE-PCR data [19], which could not distinguish capped from uncapped RNA. Notably, the 5’ ends of the capped RNAs are at positions shown earlier to be too far downstream to permit formation of an R domain needed for replication by reverse transcription [19].

In a complementary experiment, the uncapped transcripts, which originate only from TATA1, were cloned and sequenced from total RNA of embryo and callus (Figure 3C). The start sites of these RNAs for callus are at nt 1351, 1379, and 1382 (numbering from Z17327). The two major bands from embryo tissue represent *BARE1* and *BARE2* RNA and respectively start at nt 1350 (numbering from Z17327) and 1682 (numbering from AJ279072). All start sites corresponded to those found in the earlier RACE-PCR data for TATA1 in *BARE1* and *BARE2* [19].

### Both Spliced and Unspliced Capped TATA2 RNAs Are Polyadenylated

As described above, TATA2, but not TATA1, transcripts from both *BARE1* and *BARE2* are capped; those from *BARE1* are spliced about half the time and *BARE2* transcripts are not spliced. Whereas translated cellular RNAs are generally both capped and polyadenylated, plant viral RNAs and most positive-strand RNA viruses are translated not only without caps but also without poly(A) tails [30]. To clarify the situation for *BARE*, polyadenylated RNAs isolated from leaf and callus were subjected to RLM-PCR diagnostic for the presence of caps and then the PCR products sequenced. Caps were present in both *BARE1* and *BARE2* products from the polyadenylated RNA fraction; these transcripts start only after TATA2 (Figure 3D). In control experiments, the 5’ adapters were directly ligated to polyadenylated RNA from embryo and callus. The reactions yielded no product, indicating that no *BARE* RNA was simultaneously polyadenylated and uncapped.

### Polyadenylated BARE RNA Is Polyribosome-Associated

Given the multiple *BARE1* and *BARE2* RNA species, we investigated which pool is translated by examining RNA in polyribosomal translation complexes. As described above, the *BARE2* transcripts neither express Gag nor are spliced, raising the question of whether they are nonetheless present among the polyribosomal RNAs. Polyribosomes were isolated; the 28S and 18S ribosomal RNAs were effectively concentrated into the pellet (Figure 4B). The supernatant retained mainly the small RNAs (mostly tRNAs), indicating the presence of the polyribosomes in the pellet. Capped RNA was detected by 5’ RLM-RACE using a primer matching both *BARE1* and *BARE2* (Figure 4B). *BARE1* and *BARE2* RNAs were distinguished by sequencing and found in polyribosomes of leaf, callus and embryos, the three tissues investigated. Sequencing showed the start sites at nt 1689 (accession Z17327) for *BARE1* and 25nt downstream of TATA2 for *BARE2* (AJ279072) as before [19]. For comparison, cloning and sequencing of the uncapped RNA products in total RNA revealed that TATA1 transcripts start at position nt 1351 in embryos and 1412 in callus as described earlier [19].

To look at the relative abundance of *BARE1* and *BARE2* transcripts in the polyribosomes, specific primer pairs were used. The *BARE2* polyribosomal transcripts are more abundant than those of *BARE1* (Figure 4D), corresponding to earlier results for the same barley cultivar (Bomi): *BARE1* and TATA2 transcripts were shown to represent respectively 32% and 6–25% of the total *BARE* pool [16,19]. Hence, the lack of a translatable *gag* domain does not appear to interfere with the *BARE2* transcripts either being capped or forming polyribosomes and suggests that *BARE2 pol* is translated, even if *gag* cannot be, by either ribosome scanning or internal entry.

The presence of the spliced *BARE1* transcript that codes only for Gag raises the expectation that if the spliced form contributes to production of Gag it, too, should be polyadenylated and associated with polyribosomes. The total RNA from callus was used as a template for RT-PCR using primers near the splice junction; both spliced and unspliced forms were present (Figure 3E). The pellet containing the polyribosomes also contained the spliced form, at a proportion of the total *BARE* pool similar to that expected. The data show that the spliced form is not differentially excluded from polyribosomes, although some of the unspliced form remains in the supernatant. In order to examine polyribosomal *BARE* RNA for polyadenylation, total RNA was first isolated from the polyribosomes and two rounds of 3’ RLM-RACE carried out, first to select for the 3’ LTR segment of the LTR (Figure 4A) and then for a poly(A) tail. The PCR products were sequenced; the polyadenylated, polyribosomal *BARE* transcripts shared their 3’ termini with those in the polyadenylated *BARE* population in total RNA [19].

### Non-Polyadenylated *BARE* RNA Is Packaged into VLPs

**Figure 5:**
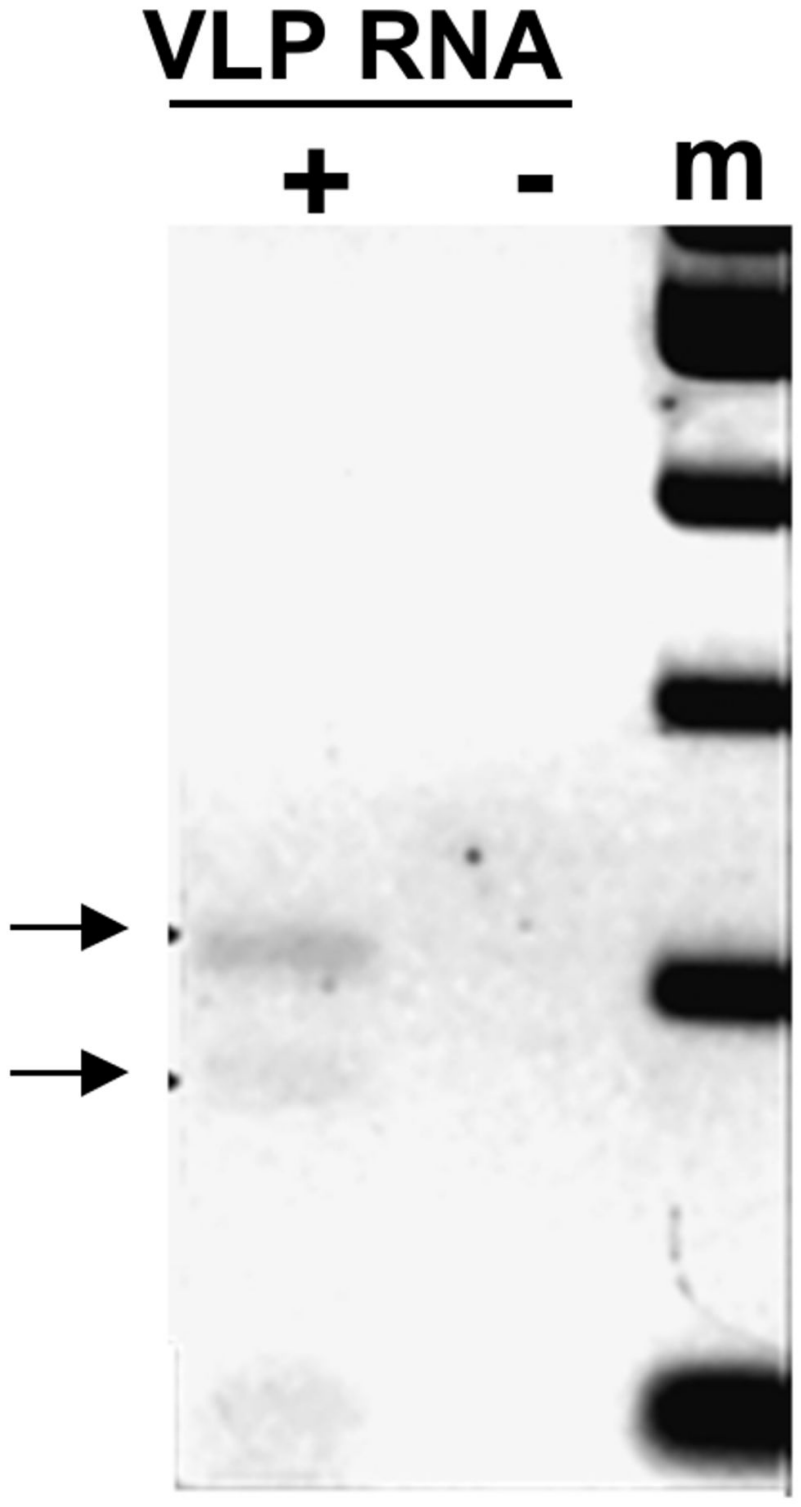
*BARE* transcripts in VLPs. Electrophoresis of 3’ RLM-RACE PCR reactions, performed on purified VLP fractions 9-11 [18]. The amplified products (arrows, 220 bp and 180 bp) represent two of the transcript groups seen earlier in total RNA, distinct from those in poly(A) [19]. Forward nested primers are RLM1 and RLM2; primer E2146, which matches the ligated linker sequence, was used as the reverse primer. 100 bp ladder (m) is shown.

The TATA1 transcripts, as shown above, are not capped, spliced, or polyadenylated. Because only they contain the R domain, if *BARE* is being replicated then TATA1 transcripts should be packaged into VLPs. To investigate this, we isolated VLPs and examined the ends of RNAs associated with them. The RNA was isolated from the pooled and purified VLP fractions 9-11 described previously [18], and used as the template for 3’ RLM-RACE (Figure 5). The sequenced products reveal that only non-polyadenylated RNA is packaged in VLPs. Sequences of the packaged RNAs have the previously described end points that are expected of transcripts initiated by TATA1 and not TATA2 [19]. Furthermore, the RNA sequences include TATA1 transcripts of *BARE2*, which is not able to produce the Gag of the VLPs into which its transcripts are packaged. This clearly demonstrates the parasitism of *BARE2* on *BARE1*.

## Discussion

**Figure 6:**
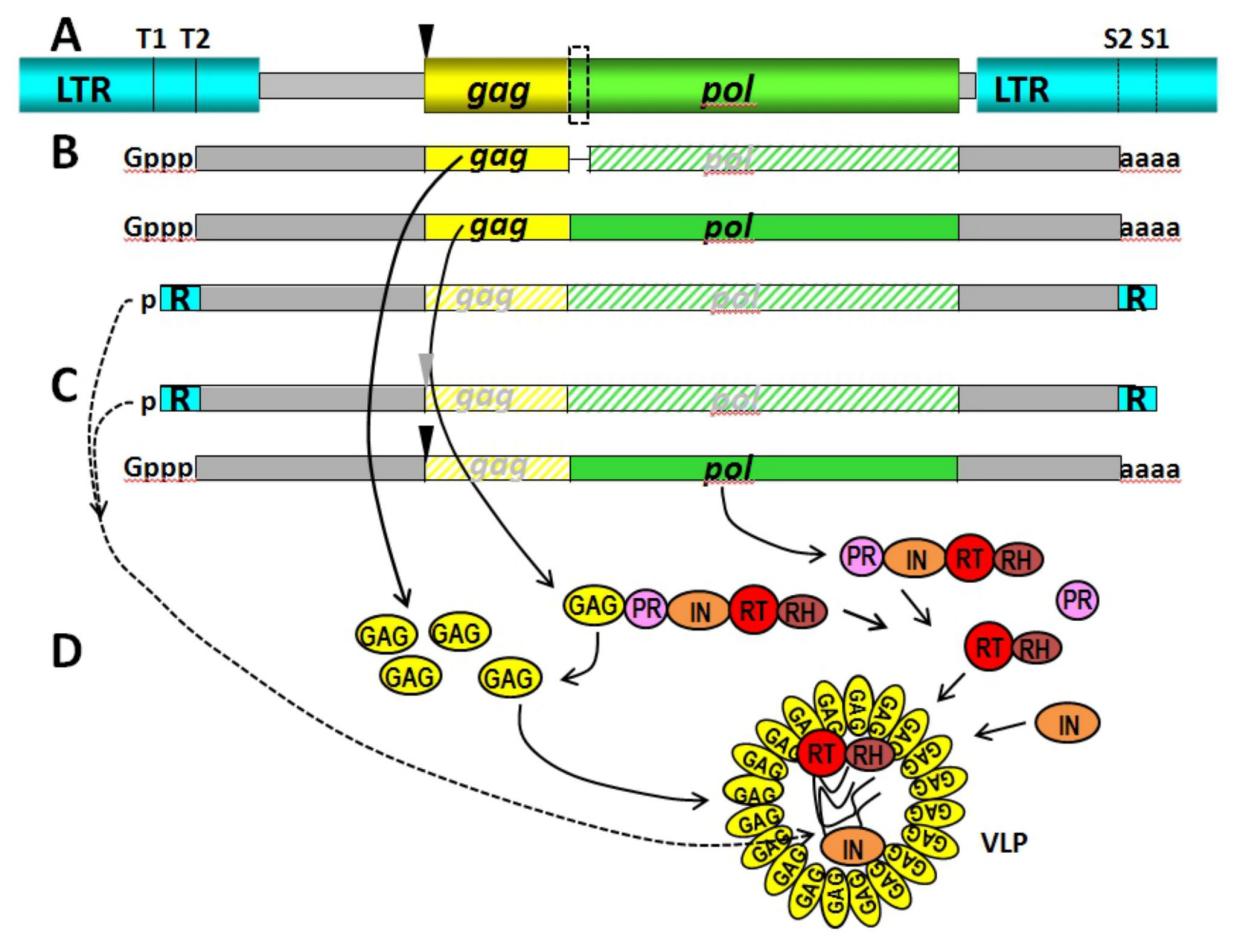
Schematic model of *BARE* RNA expression, translation, and replication. **A**. *BARE* retrotransposon, drawn to scale, showing: 5’ LTR (turquoise), including the positions of TATA1 (T1) and TATA2 (T2); untranslated leader (gray box); *gag* (yellow), encoding the capsid protein Gag; deletion in *BARE2* (black inverted triangle), which ablates *gag* start codon; *pol* (green), encoding aspartic proteinase (PR), integrase (IN), and the reverse transcriptase – RNase H complex (RT-RH); the alternatively spliced intron (dashed box), which generates a frameshift that knocks out *pol* expression in *BARE1*; 3’ untranslated region (gray box); 3’ LTR (turquoise), including termination site for transcripts from TATA2 (S2) and from TATA1 (S1). **B**. Transcripts from *BARE1*, including the alternatively spliced capped (Gppp) and polyadenylated (aaaa) RNA from TATA2 and the uncapped non-polyadenylated RNA from TATA1, the latter which have the terminal repeats (R, turquoise boxes) needed for replication into cDNA. Unexpressed ORFs are shown as hatched boxes labelled in gray C. Transcripts from *BARE2*, including the TATA1 products and the capped and polyadenylated TATA2 products, which cannot express *gag*. **D**. Mapping of the formation of the translation products from the various RNAs, including: Gag from *BARE1*; the polyprotein from *BARE1*, which is cleaved by PR into functional units (GAG, yellow; PR, violet; RT-RH (red and brown). A schematic representation of the assembly of the components into the virus-like particle (VLP) is shown, into which the TATA1 transcripts together with RT-RH and IN are packaged.

Retrotransposon and retrovirus transcripts serve two distinct roles: as templates for the proteins needed for their own replication; as genomic RNA, which is first packaged into capsids comprised of Gag, its own translation product, and then later destroyed during its reverse transcription into cDNA. We earlier showed that retrotransposons *BARE1* and its parasitic relative *BARE2*, which cannot synthesize its own capsid protein, produce two sets of transcripts; one from each of the two TATA boxes in the LTR [19,31]. We also showed that only a minority of the *BARE* transcripts, 15 to 25%, were polyadenylated, although neither was transcript processing further examined nor the reason for the incomplete polyadenylation found. Here, we have uncovered a replication system whereby *BARE1* and *BARE2* encode distinct classes of RNAs to serve the two disparate functions, one for translation and the other as the genomic RNA destined to be reverse-transcribed into cDNA (Figure 6). The results are reminiscent of the distinct pools for translation and reverse translation purportedly formed by the MLV retrovirus [13], rather than the single pool of HIV [14].

The first RNA pool, transcripts from TATA2 of both *BARE1* and *BARE2*, is capped, polyadenylated, and polyribosome-associated. These features indicate that TATA2 RNA serves for translation of the protein products of *BARE*. Earlier, we had shown that reporter gene expression driven by the *BARE* LTR is dependent on the presence of TATA2 and not TATA1 [19,31], strongly suggesting that all translated *BARE* proteins are derived from TATA2. Capping and polyadenylation have not been well investigated for LTR retrotransposons; *Ty1* and *copia* of Superfamily *Copia* are translated from capped RNA [32,33] while *Idefix* of superfamily *Gypsy* exploits both cap-dependent and independent mechanisms [34]. Retroviruses, which are likely derived from *Gypsy* retrotransposons [3], produce capped transcripts but appear to exploit both cap-dependent and ‑independent translation [35]. Here, the presence of native polyadenylated, capped TATA2 transcripts in the polyribosome “translatome” is the clearest indication of active *BARE1* translation in the tissues examined [36,37]. Interestingly, the TATA2 transcripts of *BARE2* are also capped, polyadenylated and polyribosome-associated, even if the conserved *BARE2* deletion abolishes the start codon of the ORF and thereby translation of Gag [16]. However, the subsequent AUG start codon at the end of *gag* could make the rest of *BARE2* translatable.

Translation of retrotransposons and retroviruses raises a major challenge: balancing the stoichiometry of several gene products expressed by a single promoter. The structural Gag is needed in greater abundance than the enzymes of *pol*; alteration of the ratio interferes with retroviral infectivity [38] and retrotransposon mobility [39]. Most retroviruses use -1 frameshifting to yield two reading frames, Gag and Gag-Pol [35] and most Superfamily *Gypsy* elements use +1 frameshifting [9,40]. In contrast, sequence analysis suggests that the overwhelming majority of *Copia* retrotransposons such as *BARE* encode Gag and Pol as one ORF [9], although *Ty1* uses a +1 frameshift [41].

The problem of producing enough Gag is especially acute for *BARE* for several reasons: *BARE2* lacks its own Gag [16], yet *BARE2* comprises about 68% of the *BARE* transcripts [16]; the TATA2 transcripts, which are the only ones found in the polyribosomes, comprise on average 15% of the transcripts. Despite the presence of the large pool of uncapped TATA1 *BARE* transcripts, the *BARE* retrotransposons appear not to exploit cap-independent translation on these transcripts as does HIV [29] and many plant viruses [28]. Hence, *BARE* transcripts able to express Gag amount to only 4.8% of the total. Furthermore, sequence analysis of *BARE* likewise had shown a single ORF for Gag and Pol [17]. However, here we show that, of the *BARE1* TATA2 products, about half are spliced so as to express only Gag, even if the spliced form represents only 2.4% of the total *BARE* transcript pool. Immunoblotting produces much stronger signals for Gag than for IN from barley protein extracts, consistent with the actions of a mechanism to increase the relative proportion of Gag [18].

These facts together suggest that splicing in *BARE1* may serve to increase the content of Gag compared to Pol. For *copia*, the spliced sub-genomic RNA also is present in about equal amounts with the full-length transcript [10] yet the Gag product is more abundant than Pol. The *copia* Gag RNA is translated about ten-fold more efficiently than the genomic RNA [10]. The enhancement in *copia* may be due to removal of a 2.9 kb of Pol sequence; in *BARE*, only 104 nt is spliced out to create a stop codon. The role of this domain and the translational efficiency of the spliced *BARE1* RNA are currently under investigation.

Splicing of retrotransposon transcripts in plants is known in a few other cases. The *env* sub-genomic RNA in the retroviral-like clade of superfamily *Gypsy*, which includes *Bagy*2, results from splicing [42]. Two other *Gypsy* elements, *Ogre* and *CRR*, also splice transcripts; *Ogre* splices between ORFs [20] whereas *CRR* splices to remove *rt* and create two ORFs. For retroviruses such as MMTV, expression of Gag actually requires suppression of splicing [43]. The BARE splicing reported here is the only case demonstrated in superfamily *Copia* aside from that of *copia* itself and the only one whereby a small, alternative intron within part of the *gag* ORF results in a nearly full-length sub-genomic RNA encoding just a single protein. It remains to be seen if the strategy employed by *copia* and *BARE* is general among related retrotransposons.

In contrast to translation, for replicative competence retrotransposons must avoid the packaging, reverse transcription, and integration of spliced RNA. Several lines of evidence support the view that *BARE* replicates only TATA1 transcripts, which are not spliced. First, only TATA1 transcripts contain the R domain, which is necessary for strand switching during reverse transcription [19]. Second, DNA copies of the spliced RNA, produced by TATA2, cannot be amplified from barley genomic DNA. Third, analyses of the *BARE* LTR demonstrated that TATA2 was sufficient to give full reporter expression but TATA1 alone gave none [19,31]. The strongest argument that the uncapped, un-polyadenylated TATA1 transcripts serve as the sole templates for *BARE1* cDNA synthesis, in addition to the exclusive occurrence of the R domain in them, is their presence (and the contrasting absence of TATA2 transcripts) in VLPs. It is within the VLPs that transcripts are replicated into cDNA, which is then transported back to the nucleus for integration. TATA1 transcripts of the parasitic *BARE2*, which cannot produce its own Gag, are likewise packaged into VLPs. This is consistent with the conserved dimerization and packaging signals in *BARE2* and with the predominance of *BARE2* over *BARE1* in the genomes of barley [16].

For LTR retrotransposons other than *BARE*, fairly little is known about the nature of the packaged RNA. The yeast *Ty1* is thought to package uncapped RNA, although also capped RNA may be present [44]. Although the *BARE* TATA1 transcripts were not detected with caps, they could be initially capped and then very efficiently decapped. Intriguingly, *Ty1* mRNA, Gag, and VLPs co-localize to P-bodies, components of which enhance retrotransposition [45]. The P-bodies, moreover, are sites of RNA decapping in eukaryotes [46,47]. At a minimum, TATA1 and TATA2 RNAs appear to follow very different pathways regarding RNA processing and turnover.

In summary, the deceptively simple structure of the single ORF in the *BARE* retrotransposon [17], compared with the multiple ORFs of some retroviruses [48], masks a complex expression strategy (Figure 6). Uncapped, non-polyadenylated *BARE1* and *BARE2* generated from a distinct promoter are reserved for replication into cDNA. For translation, *BARE1* appears to increase production of Gag vs Pol by splicing about half of its capped, polyadenylated transcripts. However, *BARE2* parasitizes *BARE1* Gag; its replicative transcripts are packaged into *BARE1* VLPs. To our knowledge, this is first demonstration of distinct RNA pools for translation and transcription for any retrotransposon.

At present, we are exploring the role of stress, developmental stage, and tissue on the function and relative abundance of these RNA species in order to understand how replication and propagation of *BARE* is regulated. Though it remains to be seen if the expression mechanism used by *BARE* is general, elements related to *BARE* are widespread and active [49]. Together with the *Wis*-2 [50], *Angela* [51], *OARE-1* [52], *RIRE1* [53], and *SORE1* [54] families, *BARE* is part of a group of abundant and phylogenetically diverse retrotransposons of similar structure. Therefore, the replication mechanism of *BARE* may have wide relevance.

## Supporting Information

Materials S1 **An extended description of the plant materials, RNA isolation procedures, 5**’ **cap assays, 5**’ **RLM-RACE, 3**’ **RLM-RACE, as well as polyribosome RNA isolation** and **RT-PCR, are presented.**
(PDF)

Table S1 **The primers used in this work are described by their name, sequence, matching region in *BARE1* (Accession Z17327) or *BARE2* (AJ279072), their ability to amplify products from *BARE1* or *BARE2*, and their orientation.**
(PDF)
