# Supplementary material for: BARE Retrotransposons Are Translated and Replicated via Distinct RNA Pools
Source: PLoS One. 2013 Aug 6;8(8):e72270. doi: 10.1371/journal.pone.0072270 (PMC3735527; doi:10.1371/journal.pone.0072270)
Supplement: Materials S1 — An extended description of the plant materials, RNA isolation procedures, 5’ cap assays, 5’ RLM-RACE, 3’ RLM-RACE, as well as polyribosome RNA isolation and RT-PCR, are presented. (PDF) [file pone.0072270.s001.pdf]

Materials S1

BARE retrotransposons are translated and replicated via distinct RNA pools

SUPPLEMENTARY MATERIALS AND METHODS

Plant Materials and RNA Isolation

Total RNA was isolated from either barley cv. Bomi embryos after 24 hr of imbibition on filter paper, callus (cv. Kymppi, line K19, a gift of VTT Biotechnology, Espoo, Finland), or roots of 7-day-old barley cv. Bomi seedlings. The barley callus cultures were cultured in on Medium 108 as described earlier (53). The RNA was isolated with an RNeasy kit (Qiagen 74104) according to the manufacturer's instructions. The total RNA was treated twice with DNAse (DNA-*free* kit, Ambion AM1906) according to the manufacturer's instructions before further manipulation. Polyadenylated RNA was isolated on commercial polystyrene-latex particles displaying oligo-d(T) oligonucleotides (Oligotex, Qiagen 70022) according to the manufacturer's protocol.

RNA from VLPs was isolated by extraction of sucrose gradient fractions (18) by two extractions in phenol : chloroform : isoamyl alcohol (25: 24: 1), pH 4.2, a chloroform wash, and precipitation with ethanol in buffer containing 0.3 M sodium acetate. Under the isolation conditions used (10 mM EDTA), polyribosomes are disassociated (54); moreover, no ribosomal RNA was detected in the VLP fractions. The isolated RNA was DNase treated twice as above, and DNA presence controlled by PCR with *BARE*-specific primers RLM1 and E1625; no DNA contamination was found. The 3' RLM-RACE method was used as before (19), and the PCR products gel purified, cloned, and sequenced.

5' cap assays

The presence of a 5' cap was assayed by the procedure called RNA-ligase-mediated rapid amplification of cDNA ends (RLM-RACE). First, 10 µg of DNAase-treated total RNA were dephosphorylated according to the manufacturer's protocol (FirstChoice®RLM-RACE kit, Ambion AM1700). RNA having a 5' cap is protected from dephosphorylation. The 5' end of dephosphorylated, uncapped RNA cannot be ligated to a linker for RLM-RACE (described below). The treated RNA was purified

by phenol-chloroform extraction and then ethanol-precipitated. To decap the RNA, it was then further treated with tobacco acid pyrophosphatase (TAP) following the kit protocol except that 0.5 µl RNAase inhibitor (Fermentas EO0381) was added in each step. The TAP-treated RNA was purified (RNeasy ® Mini kit, Qiagen). The decapping exposed a 5' phosphate group and enabled ligation to a 5' primer for RLM-RACE. Minus-TAP reactions, in which the substrate is not decapped and cannot be ligated, were carried out to establish that the experiments detected products that were from 5' RACE. RNA was also ligated without phosphatase and pyrophosphatase treatments to assay for the presence of uncapped RNA via RLM-RACE (described below); capped RNA cannot directly be ligated to an adapter.

5' RLM-RACE

Either total RNA or polyribosomal RNA (isolated from polyribosomes as described below) was ligated with an RNA oligonucleotide (described in Table S1) in a 50 µl ligation reaction containing 7.2 µg dephosphorylated CIP and TAP treated total RNA, 5 µl 10 x ligase buffer, 1 µl RNAsin, and 3 µl RNA ligase (2U/µl) at 37°C for 3.5 hr. Another parallel ligation reaction was set up with the treated RNA replaced by same amount of untreated total RNA to assay for uncapped RNA. These two ligation reactions were purified (RNeasy® Mini kit Qiagen) and then used for cDNA synthesis. The cDNA synthesis was primed with primer ATGA (Table S1), which anneals to the RNA at a position corresponding to the 5' end of *gag*. The first-strand reverse transcription reactions were carried out using MMLV H^{-} reverse transcriptase (Superscript GIBCO-BRL) according to the manufacturer's instructions. The control reactions lacking either reverse transcriptase, primer, or both generated no products. In order to investigate the 5' end structure of both *BARE1* and *BARE2* from the same reaction, the cDNA synthesis primer 81567, which is positioned at the integrase domain and binds to both *BARE1* and *BARE2* was used instead of ATGA, which is specific to *BARE1*.

Following cDNA synthesis, PCR amplification was carried out on 1 µl cDNA template per reaction. For *BARE1* detection, two nested reactions were made. The first used the 5′ RACE primer (Table S1) matching the RNA oligo and the retrotransposon-specific primer PBS (Table S1); the reaction was carried out for 25 cycles (30 sec at 94°C, 30 sec at 65°C, 1 min at 72°C). One µl of the first reaction was then used in the second reaction, which consisted of 35 cycles using the same

program as for the first reaction, but with the *BARE*-specific primer E1625 (Table S1) and the 5′ RACE primer. When the cDNA was primed with primer 81567 to investigate if *BARE2* is also capped, the first PCR amplication reaction used this primer and the 5’ RACE primer, and the second used primers PBS and 5’RACE. For detection of uncapped RNA, the 5’ RACE reaction was carried out as above but the RNA was ligated directly to the RNA linker without the decapping treatment. Nested PCR was then carried out using the 5’ RACE and PBS primers in the first reaction and the primers 5’ RACE and E1625 for callus RNA in the second PCR. For embryo RNA, the primers 5’RACE and uncappTA1 (Table S1) were used in the second PCR, because E1625 is not suitable for this tissue.

3’ RLM-RACE

To examine polyadenylation of polyribosome-associated RNA (isolated from polyribosomes as described below), 500 ng of RNA was first ligated with phosphorylated DNA oligo E2147 (Table S1) at room temperature overnight and the reaction products purified with an RNeasy® Mini kit (Qiagen). Nested RT-PCR was then carried by with primers E2146 (Table S1; matching E2147) and RLM1 (Table S1), followed by primers E1820 (Table S1), in order to amplify from a poly(A) tail, and RLM2 (Table S1). Primers RLM1 and RLM2 are positioned upstream of TATA1, which is in the U3 region and therefore not found in the 5’ LTR region of retrotransposon transcripts, in order to amplify specifically from the 3’ region. The polyadenylation status of RNA packaged inside of VLPs was investigated using the same method and sets of primers.

Polyribosome isolation and RT-PCR

Approximately 3 g powder was thawed in polyribosome extraction buffer (0.2 M sucrose, 0.2 M Tris-HCl pH 8.5, 0.4 M KCl, 35 mM $MgCl_2$, 25 mM EGTA, 10 mM DTT) and the mixture gently homogenized. The mixture was centrifuged at 2000 g for 5 min at 4^oC. The supernatant was adjusted to 1 % (w/v) Triton X-100 and centrifuged at 20 000 x *g* for 20 min at 4^oC. The supernatant was collected and brought to 400 mM KCl, then incubated for 10 min at room temperature. It was then layered onto 10 % - 50 % sucrose gradients prepared in polyribosome buffer and ultracentrifuged at 4^oC for 4 h at 36 000 rpm in a Sorvall TH-641 rotor. Twelve 1-ml fractions were collected using a fraction collector and the absorption of the gradient

at 260 nm was monitored. The RNA was extracted from the sample before ultracentrifugation and from the supernatant and pelleted following ultracentrifugation as for the RNA in VLPs. The extracted RNA was then treated with DNase (DNase-free kit, Ambion) twice before RT-PCR.

For detection of *BARE2* RNA associated with polyribosomes, 1 µg of DNAase treated RNA from the polyribosome pellet was reverse-transcribed into cDNA as above using primer AP4, which matches both *BARE1* and *BARE2,* followed by a second amplification with the *BARE2*-specific primers 1965 (Table S1) and 1966 (Table S1), or the *BARE1*-specific primers gag5 (Table S1) and AP4 (Table S1) described above.

REFERENCES

18. Jääskeläinen M, Mykkänen AH. Arna T, Vicient C, Suoniemi A., Kalendar R, Savilahti H, Schulman AH (1999) Retrotransposon *BARE*-1: Expression of encoded proteins and formation of virus-like particles in barley cells. Plant J. 20: 413-422.
19. Chang W, Schulman AH (2008) *BARE* retrotransposons produce multiple groups of rarely polyadenylated transcripts from two differentially regulated promoters. Plant J. 56 40 - 50.
55. Salmenkallio-Marttila M, Kauppinen V (1995) Efficient Regeneration of Fertile Plants from Protoplasts Isolated from Microspore Cultures of Barley (*Hordeum vulgare* L.). *Plant Cell Rep.* 14: 253-256.
56. Dutko JA, Kenny AE, Gamache ER, Curcio MJ (2010) 5' to 3' mRNA decay factors colocalize with the Ty1 Gag and human APBEC3G and promote Ty1 retrotransposition. J. Virol. 84: 5052-5066.
