# Supplementary material for: BARE Retrotransposons Are Translated and Replicated via Distinct RNA Pools
Source: PLoS One. 2013 Aug 6;8(8):e72270. doi: 10.1371/journal.pone.0072270 (PMC3735527; doi:10.1371/journal.pone.0072270)
Supplement: Table S1 — The primers used in this work are described by their name, sequence, matching region in BARE1 (Accession Z17327) or BARE2 (AJ279072), their ability to amplify products from BARE1 or BARE2, and their orientation. (PDF) [file pone.0072270.s002.pdf]

Table S1. Primer list.

| Primer | Sequence | Matching region in *BARE1* [1] | Amplifies from *BARE1 or BARE2* | Orientation |
|---|---|---|---|---|
| LS1 | CTGCCTCGCCCACCGGACTTGAGCTAGTGGAATTGG | 3234-3258 | 1, 2 | F |
| AP4 | CTGGTTCGGCCCACCAGATATAATGTTCATGCT(TC)AACGC | 4508-4486 | 1, 2 | R |
| 1965 | GCGAATTACGATGTAGCTTGC | 6624-6644 [2] | 2 | F |
| 1966 | GTCTCTTCATTAGTGCAGCAACTG | 7355-7332 [2] | 2 | R |
| gag5 | AAACTGCAG(A/G)TGTTGTAGAT(T/C)AATGGCTC(A/G)CG | 3380-3401 | 1 | F |
| F1593 | GGCGCTATCATTGTTAACTCTAACTC | 4697-4672 | 1 | R |
| F1594 | GCTGATAAGAAGGCGGCCAA | 4202-4221 | 1 | F |
| 81567 | GACATAAGCATCGGATCCTCAAACTTAAAG | 5387-5360 | 1, 2 | R |
| UncapTA1 | AGCCCACTAAGGGCTGGTGTG | 1453-1433 | 1 | R |
| E1625 | ACGACACCTCCGCGTTCAGC | 1908-1889 | 1 | R |
| PBS | CTACGCATGAACCTAGCTCATGATGCC | 2166-2140 | 1, 2 | R |
| ATGA | CTGGTTGGCCCACG(CT)GAGCCATT(GA)ATCTACAACA(CT)A | 3401-3377 | 1 | R |
| RLM1 | CGCAGGGTCTGCACACTTAAGGTTC | 1153-1177 | 1, 2 | F |
| RLM2 | GTTGGTTACCGAATGTTGTTCGGAGTC | 1209-1235 | 1, 2 | F |
| 5' RACE | GCTGATGGCGATGAATGAACACTG | N/A[3] | 1, 2 | F |
| E2146 | ACTCTGCGTTGTTACCACTGCTT | N/A[4] | 1, 2 | R |
| E1820 | AAGCAGTGGTAACAACGCAGAGTACT$_{30}$NA | N/A[5] | 1, 2 | |
| RNA oligo | GCUGAUGGCGAUGAAUGAACACUG | N/A[6] | 1, 2 | |

[1] In accession Z17327
[2] Matching region in Accession AJ279072
[3] Matches RNA oligo, for RLM-RACE
[4] Matches primer E1820, for 3' end mapping
[5] For making cDNA from poly(A) RNA
[6] For RLM-RACE
